# Supplementary material for: Ohr and OhrR Are Critical for Organic Peroxide Resistance and Symbiosis in Azorhizobium caulinodans ORS571
Source: Genes (Basel). 2020 Mar 20;11(3):335. doi: 10.3390/genes11030335 (PMC7141136; doi:10.3390/genes11030335)
Supplement: Supplementary file 1 [file genes-11-00335-s001.pdf]

Supporting Information

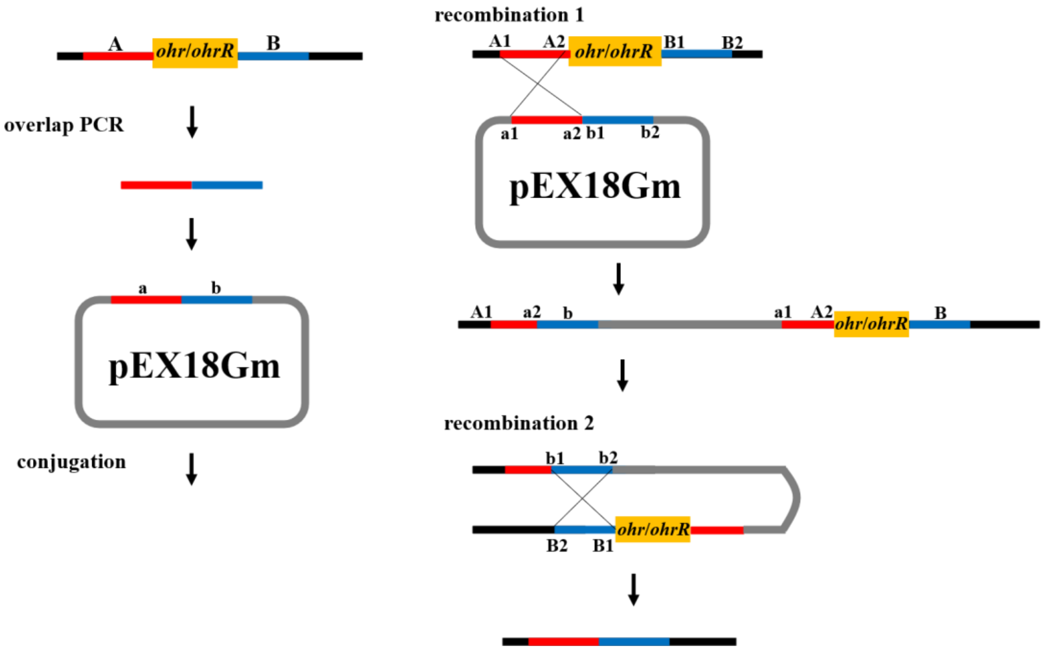

Figure S1. Schematic representation of the strategy used to construct the *ohr* and *ohrR* mutant strains by homologous recombination.

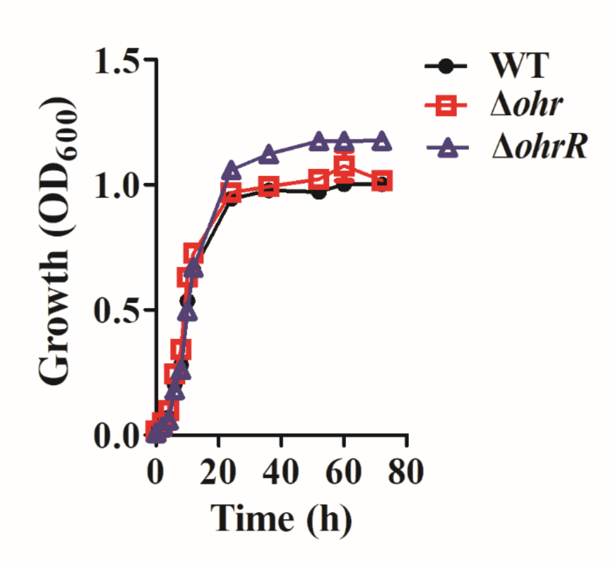

Figure S2. Bacterial growth of *A. caulinodans* WT, Δ*ohr* and Δ*ohrR* mutant strains. The cultures were grown in TY medium at 28 °C and OD_{600} was measured at different time points. Experiments were repeated 3 times.

CAAAGGGTTAGTTTTCCGTTGCGGAAGGTTGGGTGATG**TTGACG**GTGG
-35
TCTGCAAAATAAGTT**TGACACAAT**CAAATTGTGTGCTACTTAAATGGCC
-10
ATGGAAGCGGCTTGGCCGTTTCTCATCGATTTCTCAAGGCACGAGGATC
AAGCGATGTCCGTCGATGTGAAGTACACCACCAAG

Figure S3. The promoter sequence of *ohr* gene. The putative -10 and -35 promoter elements are shown in bold, and the inverted repeat motifs are indicated by arrows

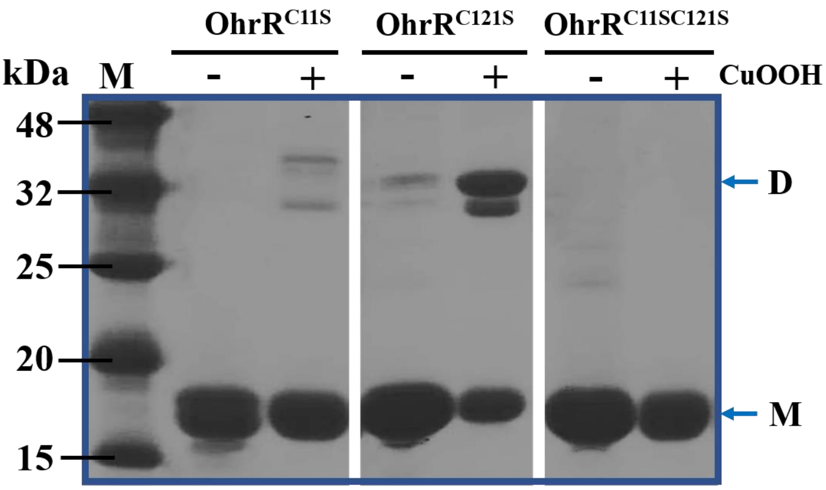

Figure S4. Investigation of intermolecular disulfide bond formation on OhrR variants by nonreducing SDS-PAGE. Samples of reduced proteins were either untreated (-) or treated (+) with CuOOH. The monomeric (M) and dimeric (D) forms of OhrR are indicated by arrows.

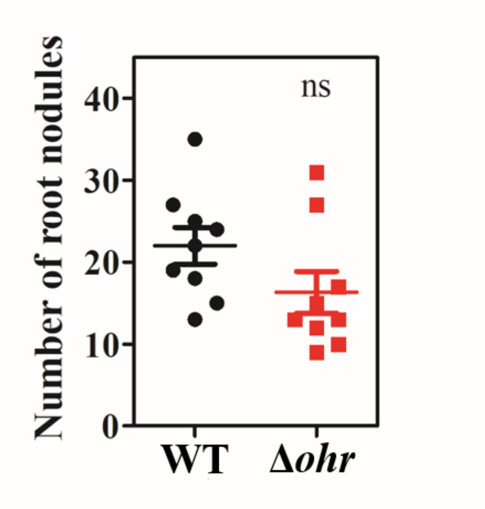

Figure S5. The number of root nodules on *S. rostrata* formed by *A. caulinodans* wild type and Δ*ohr*. Horizontal lines are the average number of nodules formed.

Table S1. Strains and plasmids used in this study.

Strains or Plasmids	Relevant characteristics	Source
A. caulinodans strains		
WT	*Azorhizobium caulinodans* ORS571, AZC0 wild-type, Amp^R	[1]
Δ*ohr*	Derivative of WT carrying an *ohr* in-frame deletion	This study
Δ*ohrR*	Derivative of WT carrying an *ohrR* in-frame deletion	This study
Δohr^c	Mutant Δ*ohr* harboring the expression construct pYC12-*ohr*	This study
Δ$ohrR^c$	Mutant Δ*ohrR* harboring the expression construct pYC12-*ohrR*	This study
E. coli strains		
DH5α	Host for cloning	[2]
SM10 λpir	Host for conjugation	[3]
BL21(DE3)	Host for protein expression	[2]
Plasmids		
pEX18Gm	Suicide cloning vector, Gm^R	[4]
pYC12	*In trans* expression vector, Gm^R	[5]
pRA302	Translational fusion vector, Spe^R	[6]
pET-28a	Protein expression vector, Kan^R	[7]

Table S2. PCR primers used in this study.

Primer	Sequence (5’-3’)[a]	Restriction sites
For deletion		
AZC2977-1	CGGAATTCTGCCCGACTGCCAGCTGACA	*Eco*RI
AZC2977-2	GCCGTCAGACGACGACGGACATCGCTTGATCCT	
AZC2977-3	AGCGATGTCCGTCGTCGTCTGACGGCGAGGCTG	
AZC2977-4	CCAAGCTTAGCCCGCCAATGTTGAAGAG	*Hind*III
AZC3555-1	CGGAATTCTCCACGCCCTTCATCTTCTG	*Eco*RI
AZC3555-2	CCTAGTCGACGGCGAAGGTGGACATGGCAAGGG	
AZC3555-3	CATGTCCACCTTCGCCGTCGACTAGGCGGCAAC	
AZC3555-4	CCAAGCTTTGCGATTGTCCACCTGATGG	*Hind*III
For *in trans* expression		
AZC2977-pYC12-F	GGGAATTCATGTCCGTCGATGTGAAGTACAC	*Eco*RI
AZC2977-pYC12-R	CCAAGCTTGCAACGGCGGACTTACTTC	*Hind*III
AZC_3555-pYC12-F	GGGAATTCATGTCCACCTTCTGCCTCGACGA	*Eco*RI
AZC_3555-pYC12-R	CCAAGCTTCTAGTCGACGGCGGCGTTCA	*Hind*III
For qRT-PCR		
16s rRNA-F	ACGGATTTCTTCCAGCAATG	
16s rRNA-R	ACCGGCAGTCCCTTTAGAGT	
AZC2977-F	ATGTGAAGTACACCACCAAGG	
AZC2977-R	CGAACAGTTGCTCGGGATT	
For transcriptional fusion		
ohr-pRA302-F	CGGAATTCTAAGGGCCGCATGAGAAAGC	*Eco*RI
ohr-pRA302-R	CCAAGCTTCCCAGTTCCTTCGGCGTGTC	*Hind*III
For site-directed mutagenesis		
OhrR-11S-1-F	GCGCTTTCGACTTCAGGTTCGGG	
OhrR-11S-1-R	GGAATAGACGGCGAAGCTCAGCAGG	
OhrR-11S-2-F	GACGACCTGCTGAGCTTCGCCGTCT	
OhrR-11S-2-R	CTAGTCGACGGCGGCGTTCAGG	
OhrR-121S-1-F	ATGTCCACCTTCTGCCTCG	
OhrR-121S-1-R	GTCCTCGGCGCTCAGGCCGATGGC	
OhrR-121S-2-F	CATCGGCCTGAGCGCCGAGGAC	
OhrR-121S-2-R	CTAGTCGACGGCGGCGTTC	
For protein expression		
OhrR-pET28a-F	CGGAATTCATGTCCACCTTCTGCCTCG	*Eco*RI
OhrR-pET28a-R	CCAAGCTTCTAGTCGACGGCGGCGTTC	*Hind*III

[a]The underline sequence is the restriction site of indicated enzymes.

References

1. Dreyfus, B.L.; Dommergues Y.R. Nitrogen-fixing nodules induced by Rhizobium on the stem of the tropical legume *Sesbania rostrata*. *FEMS Microbiology Letters*, **1981**, *10*, 313-317.
2. Chart, H.; Smith, H.R.; La Ragione, R.M.; Woodward, M.J. An investigation into the pathogenic properties of *Escherichia coli* strains BLR, BL21, DH5α and EQ1. *Journal of Applied Microbiology* **2000**, *89*, 1048-1058.
3. Ferrieres, L.; Hémery, G.; Nham, T.; Guérout, A.M.; Mazel, D.; Beloin, C.; Ghigo, J.M. Silent mischief: bacteriophage Mu insertions contaminate products of *Escherichia coli* random mutagenesis performed using suicidal transposon delivery plasmids mobilized by broad-host-range RP4 conjugative machinery. *Journal of bacteriology* **2010**, *192*, 6418-6427, doi:10.1128/JB.00621-10.
4. Metcalf, W.W.; Jiang, W.; Daniels, L.L.; Kim, S.K.; Haldimann, A.; Wanner, B.L. Conditionally replicative and conjugative plasmids carrying lacZ alpha for cloning, mutagenesis, and allele replacement in bacteria. Plasmid 1996, 35, 1–13.
5. Cao, H.; Yang, M.; Zheng, H.; Zhang, J.; Zhong, Z.; Zhu, J. Complex quorum-sensing regulatory systems regulate bacterial growth and symbiotic nodulation in *Mesorhizobium tianshanense*. *Arch Microbiol* **2009**, *191*, 283-289, doi:10.1007/s00203-008-0454-7.
6. Jiang, G.; Yang, J.; Li, X.; Cao, Y.; Liu, X.; Ling, J.; Wang, H.; Zhong, Z.; Zhu, J. Alkyl hydroperoxide reductase is important for oxidative stress resistance and symbiosis in *Azorhizobium caulinodans*. *FEMS microbiology letters* **2019**, *366*, fnz014, doi:10.1093/femsle/fnz014.
7. Dai, P.H. Multiple Sequence Elements are Involved in the Transcriptional Regulation of the Human Squalene Synthase Gene. *Journal of Biological Chemistry* **1997**, *272*, 10295-10302.
